# Profiling of subgingival plaque biofilm microbiota in female adult patients with clear aligners: a three-month prospective study

**DOI:** 10.7717/peerj.4207

**Published:** 2018-01-02

**Authors:** Runzhi Guo, Yunfei Zheng, Hao Liu, Xiaobei Li, Lingfei Jia, Weiran Li

**Affiliations:** 1 Department of Orthodontics, Peking University School and Hospital of Stomatology, Beijing, China; 2 Department of Oral and Maxillofacial Surgery, Peking University School and Hospital of Stomatology, Beijing, China; 3 Central Laboratory, Peking University School and Hospital of Stomatology, Beijing, China

**Keywords:** Microbial community, Clear aligners, 16S rRNA gene sequencing

## Abstract

**Background:**

Clear aligners are well known for facilitating oral hygiene maintenance and decreasing susceptibility to periodontal diseases as compared to conventional fixed appliances. However, few research studies focus on the subgingival microbial community during clear aligner treatment (CAT). Hence, this study investigates changes of the subgingival microbial community and its association with clinical characteristics during the first three months of CAT.

**Methods:**

Ten female patients with clear aligners were enrolled in this study. Subgingival plaque samples were obtained at three time points: before orthodontic treatment (T0), one month after orthodontic treatment (T1) and three months after orthodontic treatment (T2). DNA was then extracted from plaque samples and analyzed by 16S rRNA gene sequencing. Periodontal examinations, including plaque index (PI) and gingival bleeding index (GBI) measurements were also recorded.

**Results:**

The plaque indices (PIs) and gingival bleeding indices (GBIs) were slightly increased at T1 and T2, but no statistically significant difference was found. The alpha diversity indices, including the ACE, Chao1, Shannon indices, all showed a declining trend without significance, and a rising trend in the Simpson diversity index was observed. The weighted UniFrac distance was significantly higher at T1 and T2 compared with T0. Principal Coordinates Analysis (PCoA) demonstrated that the communities at T0 tended to cluster apart from the communities at T1 and T2. The relative abundance of the phylum *Firmicutes* and genus * Mycoplasma* was significantly increased at T0 compared with T2. There was no significant difference in the relative abundance of periodontal pathogens at the genus and species levels or core microorganisms at the genus level.

**Conclusion:**

A slightly decreasing microbial diversity with a significant change of microbial structure was found during the first three-month clear aligner treatment (CAT). However, subjects receiving clear aligner treatment were free from periodontal diseases with relatively stable levels of periodontal microorganisms and core microorganisms. Thus, our preliminary findings indicated that clear aligners induced nonpathogenic changes of the subgingival microbiome in the first three-month treatment.

## Introduction

In recent years, the increase in the number of adults seeking orthodontic treatment has led to a corresponding increase in the demand for appliances that are both more aesthetic and more comfortable than conventional fixed appliances (Weir, 2017). Clear Aligner Treatment (CAT) refers to the use of clear thermoformed plastic aligners that cover many or all of the teeth. With the development of dental materials and 3D technology, clear aligners have been applied to various malocclusion treatments (Martorelli et al., 2013). Hence, adult patients, especially female adults, who choose clear aligner treatment are increasing annually.

However, one of the common concerns during orthodontic therapy is periodontal complication, and adult patients are at a higher risk of periodontal diseases compared with adolescent patients (Needleman, Nibali & Iorio, 2015; Ong & Wang, 2002). Additionally, current evidence suggested that the plaque accumulation caused by orthodontic appliances and improper orthodontic force could alter the equilibrium of the microbial ecosystem and increase the potential for pathogenicity (Ong & Wang, 2002).

Unlike fixed appliances, clear aligners are removable and facilitate oral hygiene maintenance. As such, CAT has been suggested in orthodontic treatment plan at risk of periodontitis (Karkhanechi et al., 2013; Rossini et al., 2015). Compared with fixed appliances, clear aligners were associated with better periodontal status and decreased amounts of periodontopathic bacteria as measured by the metabolism of anaerobic bacteria (Karkhanechi et al., 2013). Although clear aligner patients have lower periodontal indices, such as bleeding on probing (BOP) and the plaque index (PLI), than fixed appliance patients, some research reported that the periodontal indices during CAT are slightly higher than those before CAT (Azaripour et al., 2015).

Periodontal disease is one of the most common bacterial diseases. Previous researchers analyzed certain suspected periodontopathogens to reflect the relationship between periodontal diseases and microbial ecology (Fujinaka et al., 2013). Yet recent research found that periodontal diseases may be caused by an obvious shift in the microbial community instead of by the changes of individual periodontopathogens (Kumar et al., 2006; Zijnge et al., 2012). Changes in the microbial community could disrupt the balance between host and microorganism, and in turn lead to periodontal disease. It is crucial to understand the composition and changes of the microbiome during orthodontic therapy to avoid the development of periodontal diseases with the use of orthodontic appliances. However, the impact of CAT on the subgingival microbiome is still unclear.

Traditional biochemical methods for microbiological identification, such as PCR and DNA-DNA hybridization techniques, are applied to analyze certain types of periodontopathogens but are limited in reflecting the shift in the microbial community. 16S rRNA gene sequencing, a next-generation sequencing method, can analyze the microbial composition and community structure, providing an unprecedented amount of information. This high-throughput and cost-effective technique simplifies the exploration of the sophisticated microbial diversity (Cummings et al., 2013). Also, it has never been applied to analyze microbial change during CAT. Thus, it is essential to investigate the subgingival plaque biofilm microbiota in patients receiving CAT using 16S rRNA gene sequencing. In this study, we conducted a preliminary investigation of the microbial community changes and its association with clinical characteristic at the early stage of CAT.

## Material and Methods

### Selection of study group

Ten female patients with clear aligners were selected into our study. All of the included patients were recruited from the orthodontic department of Peking University School and the Hospital of Stomatology. All patients gave written informed consent to participate in this study. The study was approved by the Peking University Biomedical Ethics Committee (Beijing, China) (PKUSSIRB-2012063).

The patients were included according to the following criteria:

(1) 18 to 40 years old with mild to moderate tooth crowding.
(2) No missing first molars or first central incisors. No crowns or fixed bridges.
(3) No periodontitis. Before appliance placement, patients with a periodontal probing depth of less than 3 mm and no periodontal attachment loss were included.
(4) One month before the baseline examination, all included patients underwent subgingival ultrasonic scaling.
(5) Good oral hygiene and no smoking.
(6) No intake of antibiotics or hormones one month before joining the study.
(7) No pregnancy or systemic disease.
(8) Patients with ceramic brackets or lingual brackets were excluded.
(9) The gingival margin of the clear aligner is above the gingiva.

### Subgingival plaque sampling and periodontal examination

Subgingival plaque samples and periodontal examination results were obtained at three time points: before orthodontic treatment (T0), one month after orthodontic treatment (T1) and three months after orthodontic treatment (T2). At each time point, sample collection and periodontal examinations were performed before any orthodontic treatment. Eight teeth (maxillary first molars, maxillary first central incisors, mandibular first molars and mandibular first central incisors) were selected as the index teeth in our study. No periodontal treatment and mouthwash were allowed within the first three months. Oral health instructions are received by the included patients.

The clinical parameters, including plaque index (PI) and gingival bleeding index (GBI), were recorded by an experienced periodontist. The Quigley-Hein Plaque Index was used to assess the plaque coverage (Kilian et al., 2016). The GBI was measured by inserting a CP-12 probe horizontally into the interproximal papilla with a probing force of approximately 0.75 N. The scores of clinical parameters for each patient were determined by the mean measurements of index teeth.

After isolation with sterile cotton rolls and removal of supragingival plaque, subgingival plaque samples were collected with a sterilized periodontal curette. The samples were collected from the labial or buccal surfaces of index teeth and were pooled together into a 1.5-ml microcentrifuge tube containing 1.0 ml of sterilized saline water. The samples were stored immediately at −80 °C.

### DNA extraction, PCR amplification and 16S rRNA gene sequencing

DNA was extracted from subgingival plaque samples using a commercial bacterial DNA mini kit (Tiangen Biotechnologies, Beijing, China), following the manufacturer’s instructions with an additional lysozyme treatment. The final quality and quantity of DNA was measured using a NanoDrop ND1000 spectrophotometer (NanoDrop Technologies, Inc.). The v3-v4 hypervariable regions of tnhe 16S rDNA were amplified using PCR. Library preparation and sequencing was performed on an Illumina MiSeq Platform at the Biomarker Institute (Biomarker Institute, Beijing, China). The sequences obtained have been submitted to the NCBI Short Reads Archive database under Accession Number SRP114894.

### Sequence processing and statistical analysis

The sequencing data were processed using the Mothur and the Quantitative Insights Into Microbial Ecology (QIIME) pipeline. After raw sequences were trimmed and filtered, the remaining high-quality sequences with a similarity threshold of 97% were assigned to the same operational taxonomic units (OTUs). Then, using UCLUST, sequences were annotated separately against the SILVA 16SrRNA reference alignment and the Human Oral Microbiome Database (Dewhirst et al., 2010; Pruesse et al., 2007). Differences in alpha diversity and beta diversity at different time points were evaluated by ANOVA. The Principal Coordinates Analysis (PCoA) using weighted UniFrac distance matrices was performed to ordinate the dissimilarity matrices, and an ADONIS test was performed to evaluate the statistical significance. The differences in relative abundance among groups were compared using the Metastats method.

## Results

### Clinical results

The clinical parameters of the included patients are presented in Table 1. The mean age of the 10 included female patients was 25.4 ± 6.2 years. According to the low PIs and GBIs at T0, all 10 patients exhibited good oral hygiene and periodontal health before clear aligner placement. The PIs and GBIs were slightly increased at T1 and T2, but no statistically significant differences were found.

**Table 1:** Clinical characteristics of patients with clear aligners. The plaque index (PI) was recorded with a score of 0–5 using the following classification: (0) no plaque, (1) flecks of plaque at gingival margin, (2) definite line of plaque at gingival margin, (3) plaque covering <1∕3 of tooth surface, (4) plaque covering >1∕3 and <2∕3 of tooth surface and (5) plaque covering >2∕3 of tooth surface. The gingival bleeding index (GBI) was recorded with a score of 0–5 using the following classification: (0) healthy gingival, no bleeding, (1) edema, change in color without bleeding, (2) bleeding without flow along gingival margin, (3) bleeding with flow along gingival margin, (4) copious bleeding, (5) severe inflammation with a tendency for spontaneous bleeding. The scores of clinical parameters for each patient were determined by the mean measurements of index teeth.

Time points	Plaque index (PI)	Gingival bleeding index(GBI)
	(mean ± s.d.)	(mean ± s.d.)
T0	0.6 ± 0.52	0.8 ± 0.79
T1	0.8 ± 0.63	1.1 ± 0.57
T2	0.7 ± 0.74	0.9 ± 0.67

**Figure 1:**
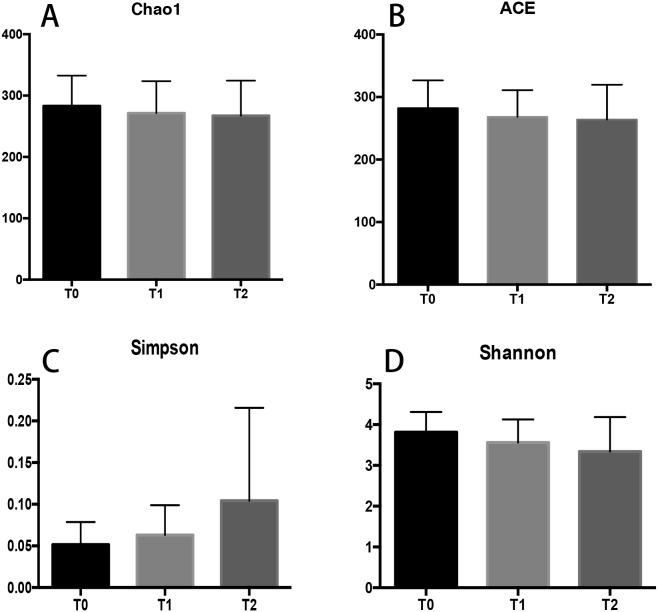
Alpha diversity indices of subgingival plaque at T0, T1 and T2. The Chao1 (A), ACE index (B) and Shannon index (D) showed a slightly decreasing trend. The Simpson index (C) at T2 was greater than that at T0 and T1 without statistical significance. All data are expressed as mean ± standard deviation.

### OTU analysis

In total, 2,212,238 valid reads were generated from 30 subgingival plaque samples with an average of 73,741 ± 2,388 (ranging from 80,135 to 55,229) reads per sample. After data processing, 1,557,064 high-quality reads were obtained with an average of 51,902 ± 1,228 (ranging from 59,878 to 38,486) sequences per sample. The average length of the reads was 440 ± 0.7 bp (ranging from 435 bp to 447 bp). After clustering all reads using a 97% similarity cutoff, we finally detected a total of 414 OTUs, with an average of 245 ± 30 (ranging from 101 to 330) OTUs per sample.

### Alpha diversity

Microbial diversity within each group was calculated and compared among the three groups. The microbial richness estimator (Chao 1 and ACE index values) showed no significant difference (Figs. 1A and 1B). The microbial community diversity estimators (the Simpson and Shannon diversity indices) showed that samples from T0 harbored slightly more diverse bacterial communities than that from T1 or T2 (Figs. 1C and 1D; *p* > 0.05). The microbial communities of samples at T0 were the most diverse, and those of samples at T2 were the least diverse.

### Beta diversity

**Figure 2:**
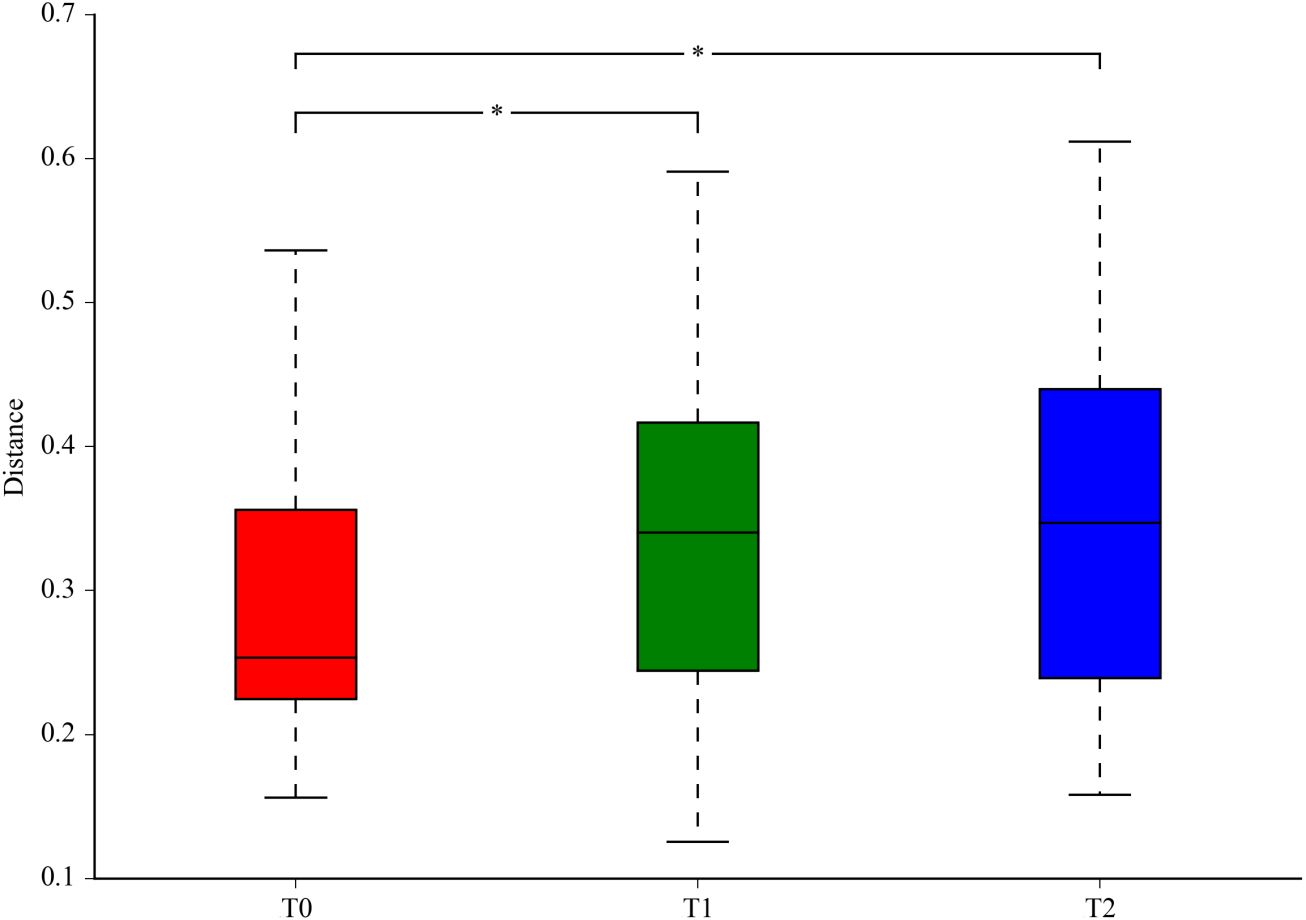
Beta diversity (the average weighted UniFrac distance value) at three different time points. Beta diversities were significantly higher at T1 and T2 compared with T0. ^∗^*P* < 0.05 by ANOVA.

**Figure 3:**
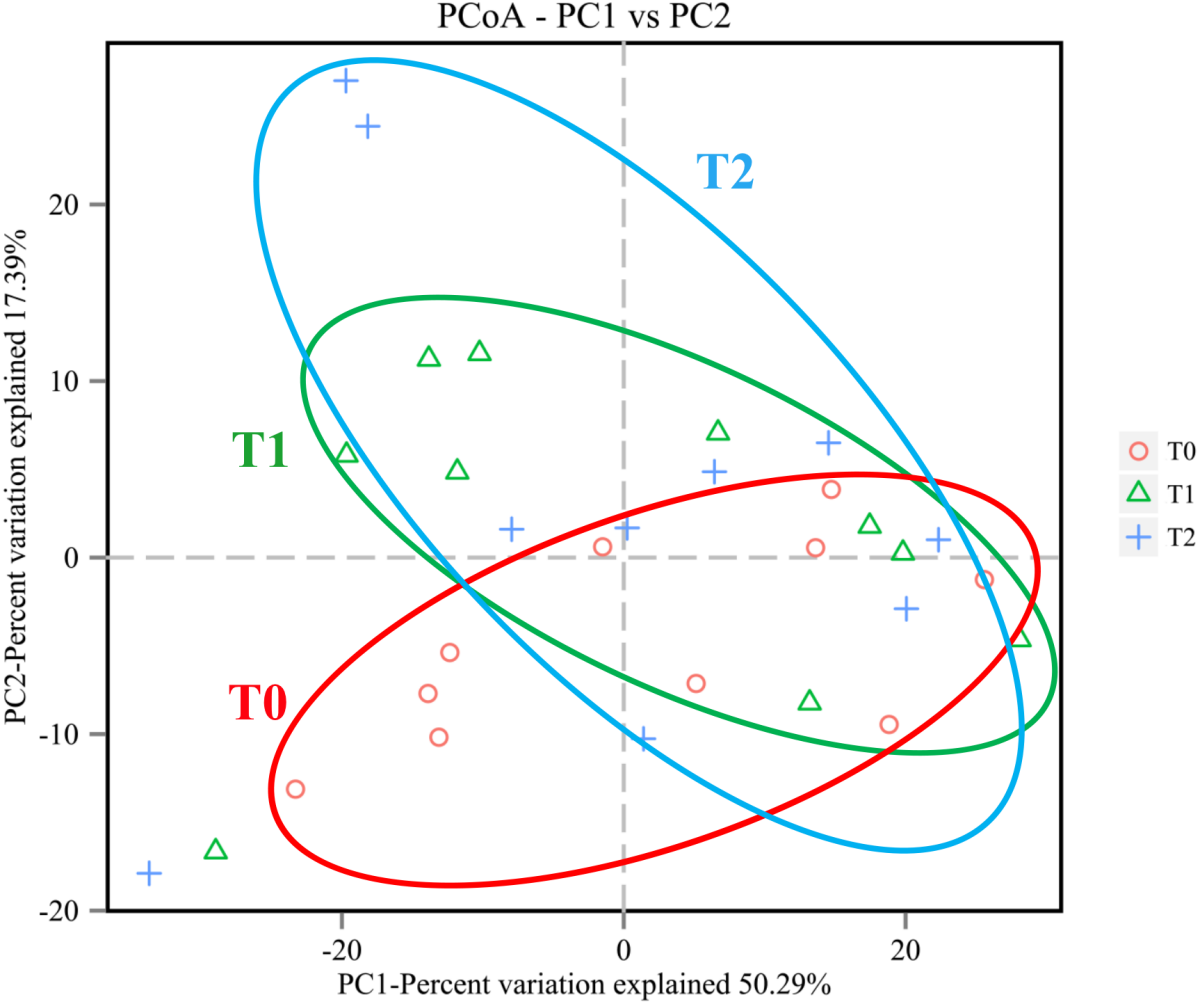
Principal Coordinates Analysis (PCoA) based on the relative abundance of OTUs identified in the subgingival plaque at T0, T1 and T2. The communities at T0 tended to cluster apart from the communities at T1 and T2 (*P* < 0.05).

The variation in microbial community structures based on weighted UniFrac distance measurements was compared at T0, T1 and T2. The variations of the beta diversity at T1 and T2 were higher than that at T0 (*p* < 0.05), and the beta diversity at T1 and T2 were more similar (Fig. 2). As shown in Fig. 3, the Principal Coordinates Analysis (PCoA) based on the weighted UniFrac distance value also showed that the communities at T0 tended to cluster apart from the communities at T1 and T2 (*P* < 0.05).

### Microbial distribution and relative abundances

The community taxonomic analysis was performed using MEGAN (Metagenome analysis software) and a phylogenetic tree was generated according to the abundance and evolutionary relationship of the microbiome (Fig. S1). To further investigate the changes of the microbial community structure, microbial distribution and relative abundances were analyzed at the phylum, genus and species levels.

#### Phyla

**Figure 4:**
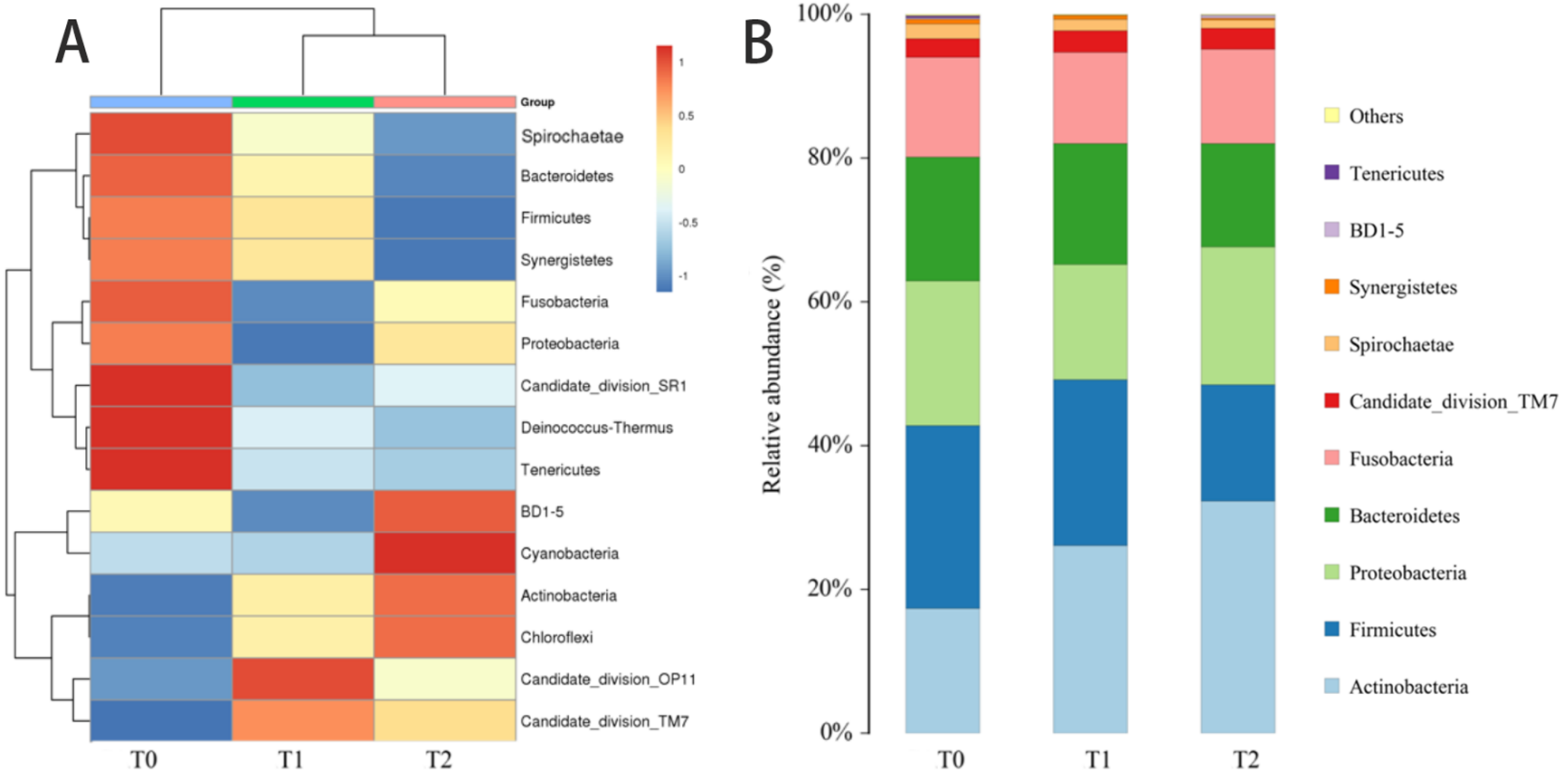
Analysis of the change of microbial structure at the phylum level. (A) Heatmap of the relative abundance of subgingival bacteria at the phylum level at three different time points. (B) Phylum-level taxon distribution at T0, T1 and T2.

**Figure 5:**
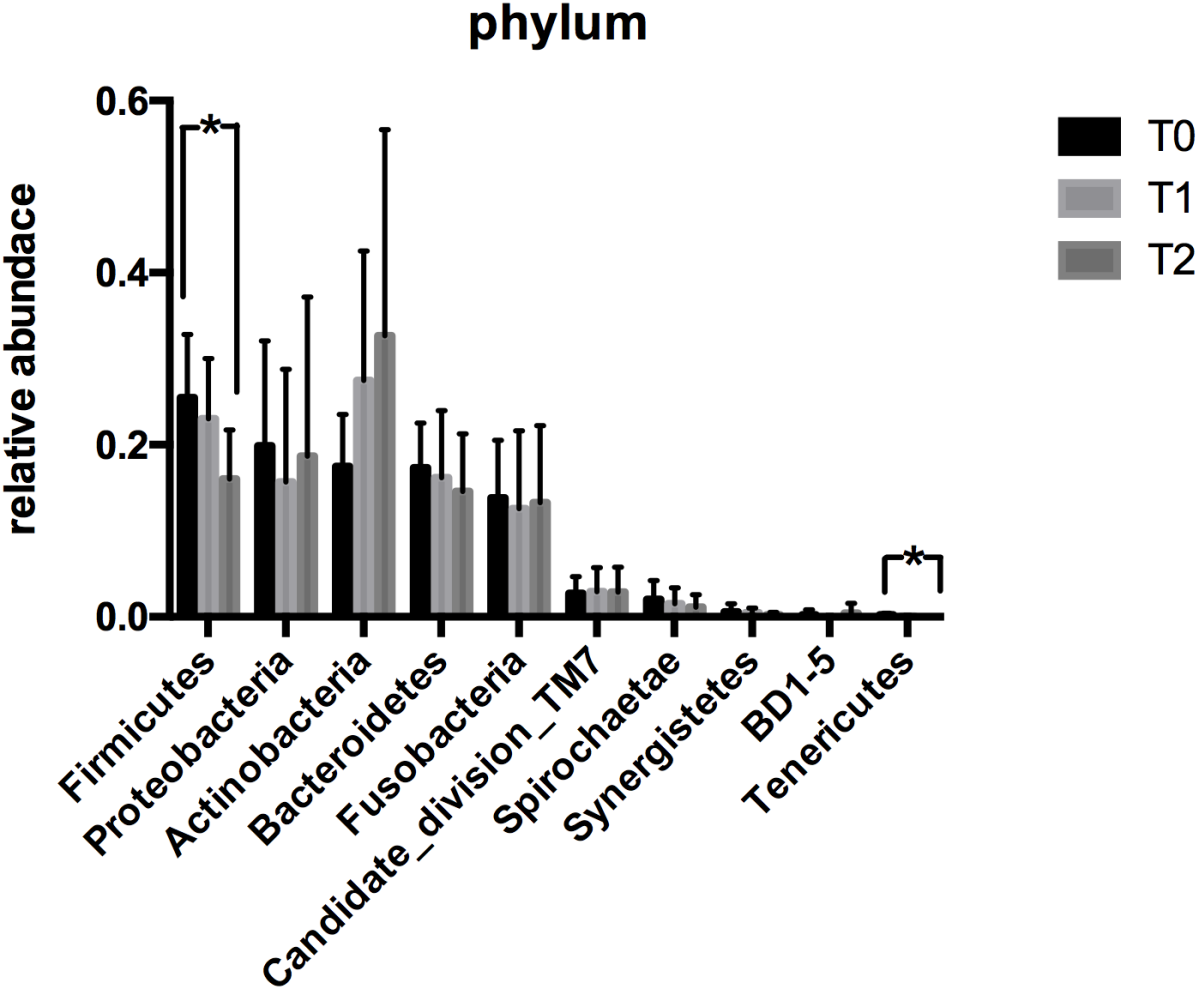
The relative abundance of the top 10 phyla. *Firmicutes* and * Tenericutes* exhibited a higher abundance at T0 compared with T2. All data are expressed as mean ± standard deviation. ^∗^*P* < 0.05 by ANOVA.

The heatmap that demonstrates the profile of microbiota at the phylum level is shown in Fig. 4A. Analyses of OTUs from the plaque samples at T0, T1 and T2 clustered within 15 bacterial phyla. The relative abundances of the top 10 phyla are illustrated in Fig. 4B. Generally, the predominant phyla of all samples were *Firmicutes, Proteobacteria*, *Actinobacteria, Fusobacteria* and *Bacteroidetes*. The most dominant phylum at T0, *Firmicutes* (25.41%), showed a significantly reduced abundance at T2 (16.15%). The changes in abundance of the remaining predominant phyla were less obvious. As shown in Fig. 5, *Firmicutes* and *Tenericutes* exhibited a higher abundance at T0 compared with T2 (*P* < 0.05).

#### Genera

**Figure 6:**
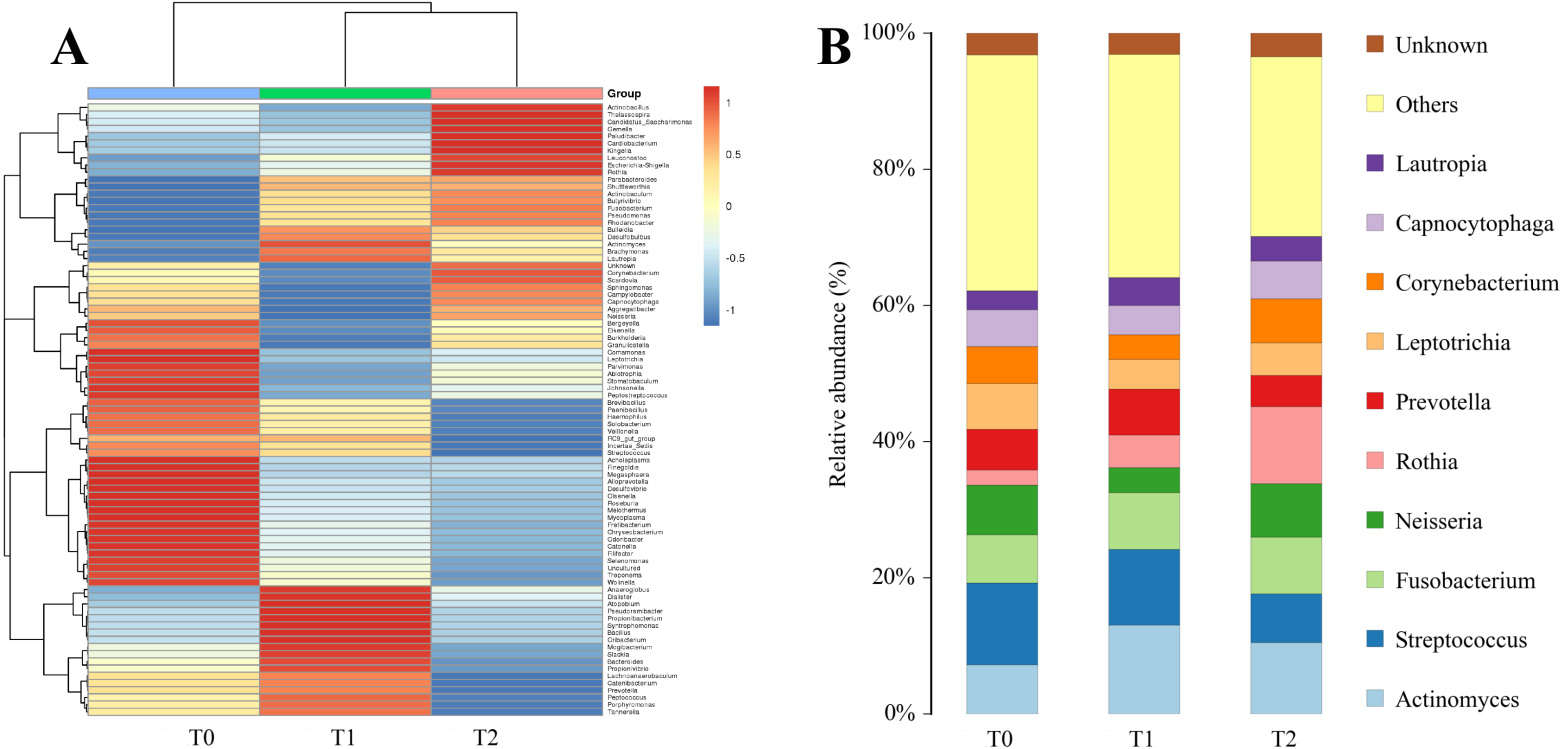
Analysis of the change of microbial structure at the genus level. (A) Heatmap of relative abundance of subgingival bacteria at genus level at three different time points. (B) Genus-level taxon distribution at T0, T1 and T2.

**Figure 7:**
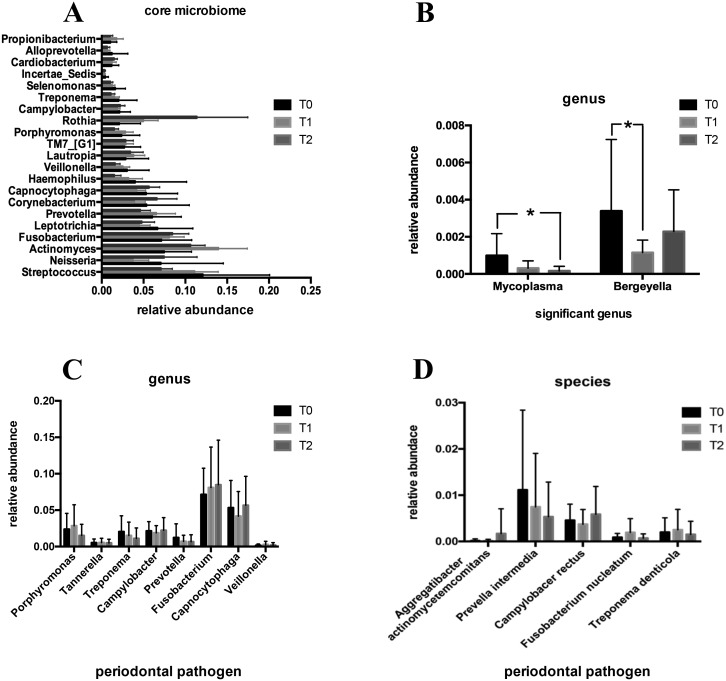
Analysis of the relative abundance of core microbiome, significant genus and periodontal pathogens. (A) The relative abundance of the core microbiome at the genus level. (B) The relative abundance of the significant microbiota at the genus level within three different time points. (C, D) The relative abundance of eight periodontal pathogens at the genus level and five periodontal pathogens at the species level within three different time points. All data are expressed as mean ± standard deviation. ^∗^*P* < 0.05 by ANOVA.

A heatmap analysis of relative abundance of subgingival microbiota at the genus level is shown in Fig. 6A. The top 85 genera were detected and compared at T0, T1 and T2. Among the detected genera, the microbial distribution of the top 10 genera is provided in Fig. 6B. It is obvious that the microbial community structure changed during the first three-month CAT. The five genera, *Streptococcus, Neisseria, Actinomyces, Fusobacterium* and *Rothia* were relatively abundant. Among these, *Streptococcus* (11.95%), *Neisseria* (7.29%) and *Actinomyces* (7.27%) showed high abundances at T0, while *Actinomyces* (13.10%), *Streptococcus* (11.09%) and *Fusobacterium* (8.34%) manifested high abundances at T1, and *Rothia* (11.36%), *Actinomyces* (10.53%) and *Fusobacterium* (8.36%) had highest abundances at T2. The relative abundances of core genera, which were defined as most commonly detected (relative abundance > 1.0%), and shared genera among groups are expressed in Fig. 7A. Among those core genera, *Rothia* and *Actinomyces* were relatively abundant at T2, with higher abundance of *Streptococcus* at T0, but all of these changes were not statistically significant. In addition to core genera, *Bergeyella* and *Mycoplasma* exhibited significant differences in abundance (Fig. 7B). The relative abundance of *Bergeyella* was significantly higher at T0 (0.34%) compared with T1 (0.11%). *Mycoplasma* showed a significantly reduced abundance between T0 (0.10%) and T2 (0.01%). Additionally, the relative abundances of eight predominant genera that could cause periodontal disease were found to have no significant difference during the first three-month CAT (Fig. 7C).

#### Species

At the species level, the periodontal pathogens investigated in our study were *Aggregatibacter actinomycetemcomitans (Aa)*, *Prevella intermedia (Pi), Campylobacer rectus (Cr), Fusobacterium nucleatum (Fn)* and *Treponema denticola (Td).* The relative abundance of *Pi* slightly decreased at T2 (0.55%) and T1 (0.80%) compared with T0 (1.15%). A slightly increased relative abundance of *Aa* at T2 (0.17%) was found. However, the changes in relative abundances of these five putative periodontal pathogens were not significantly different during the first three-month CAT (Fig. 7D).

## Discussion

There is no debate that there is a close correlation between fixed appliances and periodontal health. Many research studies have been carried out on microbial changes during fixed appliance treatment (Freitas et al., 2014; Ireland et al., 2014). Our previous research found that the level of subgingival periodontal pathogens could temporarily increase after the placement of a fixed appliance (Guo et al., 2017). However, we know little about how clear aligner treatment impacts microbiota and periodontal health. To the best of our knowledge, only one study using PCR techniques reported that patients with clear aligners had a better periodontal status and found no detectable periodontal pathogens (Levrini et al., 2015). However, 16S rRNA gene sequencing has never been applied to analyze the changes of the subgingival microbial community during CAT. Using high-throughput 16S rRNA sequencing, our study has described the change of the microbial community during the first three-month clear aligner treatment, and we found that clear aligners could affect the subgingival microbial community by decreasing the overall microbial diversity and changing the microbial structure, but the periodontal pathogens and core microbiota were less affected.

Previous research has led to the idea that clear aligners are less susceptible to periodontal disease than other fixed dental appliances. A systematic review by Rossini et al. (2015) reported that there was no statistically significant increase in probing depth during the clear aligner treatment. Hence, it is generally assumed that clear aligners have a better, or at least equal, periodontal health status compared with fixed appliances. These studies all found no significant change in the probing depth during the clear aligner treatment. It is reported that the PI and GBI were slightly higher during the CAT than that before CAT (Kumar et al., 2006). Hence, instead of periodontal disease, maybe we should be concerned about gingivitis. However, in our study, the PI and GBI at T0, T1 and T2 were not significantly increased, which indicates that the clear aligner was not susceptible to induce gingival inflammation at the first three-month treatment.

Using 16S high-throughput sequencing, a substantial improvement in our knowledge of subgingival microbial communities was achieved. Both cultured and uncultured periodontal pathogens can be detected with this method. Unlike other microbial detection techniques, such as traditional detection methods and PCR, the 16S high-throughput sequencing is able to reflect the composition and structure of the subgingival microbial community (Xiao et al., 2016). Kumar et al. (2006) used 16S rRNA gene sequencing approach to analyze the stability of subgingival microbial community and found the close relationship between shifts in microbial community and changes in periodontal health status (Levrini et al., 2015). During the orthodontic treatment, changes in the subgingival microbial community during the early stage of periodontal disease would aid in diagnosis to avoid further periodontal destruction.

A microbial community with high diversity represents a healthier and more stable status than a microbial community with low diversity that is dominated by few microorganisms (Camelo-Castillo et al., 2015). In our study, the alpha diversity indices, which reflect microbial richness and evenness, were slightly decreased overtime without statistical significance. In addition to these decreasing indices, the higher Simpson index at T1 and T2 also support a lower microbial diversity. Based on the results, a stable periodontal status was considered with a less diversity community that may not cause periodontal inflammation during the first three-month CAT.

Although the subgingival microbiota has interpersonal variability, the overall structure of the microbial community could still reflect the periodontal status. Based on weighted UniFrac distance, beta diversities were significantly higher at T1 and T2 compared with T0, indicating that a more variable community structure was observed at T1 and T2. The discrepancies of microbial community structure at three different phases were also exhibited by the relative segregation in PCoA. In addition, the heatmap and microbial distribution based on the relative abundance illustrated a more detailed comparison of the microbial structure at the genus and phylum level. Both analyses illustrated changes in the microbial community membership.

Although the microbial diversity and microbial community structure were altered, the relative abundance of most microorganisms showed no statistically significant differences at phylum, genus and species level. However, significant changes in abundance were observed in several microorganisms between time-points. At the phylum level, *Firmicutes* and *Tenericutes* were significantly more abundant at T0 compared to T2. The phylum *Actinobacteria* exhibited an increased tendency in abundance. At the genus level, we found that *Mycoplasma* and *Bergeyella* showed a significantly reduced abundance at T2 and T1, respectively. Among these, the phylum *Firmicutes* and the genus *Mycoplasma* were recognized as predominant microorganisms in the periodontal disease and decreased during the first three-month CAT. Previously, Li et al. (2014) and Abusleme et al. (2013) confirmed the association of *Firmicutes* with periodontitis. Holt, Wilson & Musa (1995) found that *Mycoplasma* may play a role in gingivitis. One explanation for the decrease in these two periodontal pathogens could be that clear aligners may facilitate in oral health maintenance. Of course, considering the less relative abundance of *Mycoplasma* at T0, another explanation for the decrease of might be bioinformatic artefacts.

Additionally, the genera of eight acknowledged periodontal pathogens were further investigated, and none were found to change in abundance significantly. At the species level, considering the limited information derived from the v3-v4 hypervariable regions of the 16S rDNA, most species cannot be discriminated. Only five putative periodontal pathogens, including *Aa*, *Pi, Cr, Fn* and *Td,* were identified. No significant changes in the relative abundance of these five putative periodontal pathogens were detected during the first three-month CAT. These results indicate that clear aligners may not specifically affect the periodontal pathogens, at least without increasing the relative abundance of periodontal pathogens, some of which was even significantly decreased.

Generally, core microbiomes are defined as the shared microorganisms which could reflect the commonality of the microbial community. The core microbiome plays a key role in periodontal health (Zaura et al., 2009). In our study, 21 genera, of which the relative abundance constituted more than 1% and were shared by all included subjects, were identified as establishing the core microbiome. Among these genera, the relative abundances of the genera *Rothia* and *Actinomyces* were higher at T2, the relative abundance of the genera *Streptococcus* was lower, but without statistically significant difference. *Rothia* and *Streptococcus* were both considered non-pathogenic microorganisms in periodontal disease (Nightingale et al., 2014). Hence, the relative stability of core microbiomes might associate with a relatively periodontal health status .

To date, how the clear aligners affect the subgingival microbial community has remained unelucidated. There are many possible factors contributing to the change of the subgingival microbial community during clear aligner treatment. Firstly, the gingival margin of the clear aligner, an apparent undercut, would cause extensive biofilm accumulation that can have an influence on subgingival bacteria. In addition, a clear aligner, a type of removable thermoplastic appliance, is prone to be adhered by the salivary film (Shpack et al., 2014). A clear aligner is not completely smooth, and the microcracks and abraded areas would facilitate bacterial adhesion (Low et al., 2011). However, the microorganisms can spread from other oral areas, such as saliva and mucosa, into the subgingival pocket (Fu et al., 2016). Hence, changes in oral microbiota in other areas caused by clear aligner could influence the subgingival microbial community.

Considering the small size of included patients and the relatively short observation time, it is essential to be aware that our findings are a preliminary investigation of the microbial community and its association with clinical parameters at the early stage of CAT.

## Conclusions

Our results revealed a change of the subgingival microbial community during the first three-month CAT. We observed a significant change of the subgingival microbial structure with a decreasing microbial diversity. Although the microbial community was affected by clear aligner treatment, the relative abundance of periodontal pathogens and core microorganisms remained stable. Hence, considering the healthy periodontal status, the microbial community affected by clear aligner is considered to be less susceptible to periodontal diseases at the early stage of CAT. Further studies with large sample size are required to demonstrate microbial community changes throughout the clear aligner treatment.

##  Supplemental Information

10.7717/peerj.4207/supp-1 Figure S1 A classification tree showing the microbial composition and abundance by MEGAN The larger the area of the pie chart at each level, the greater the bacterial abundance. Different colors represent different time points. The larger the colored sectorial area within the pie chart, the greater the bacterial abundance at the corresponding time point.

## Additional Information and Declarations

### Data Availability

The following information was supplied regarding data availability: NCBI SRA database: accession number SRR5933802.
