# Supplementary figures and images for: Profiling of subgingival plaque biofilm microbiota in female adult patients with clear aligners: a three-month prospective study
Source: PeerJ. 2018 Jan 2;6:e4207. doi: 10.7717/peerj.4207 (PMC5755484; doi:10.7717/peerj.4207)

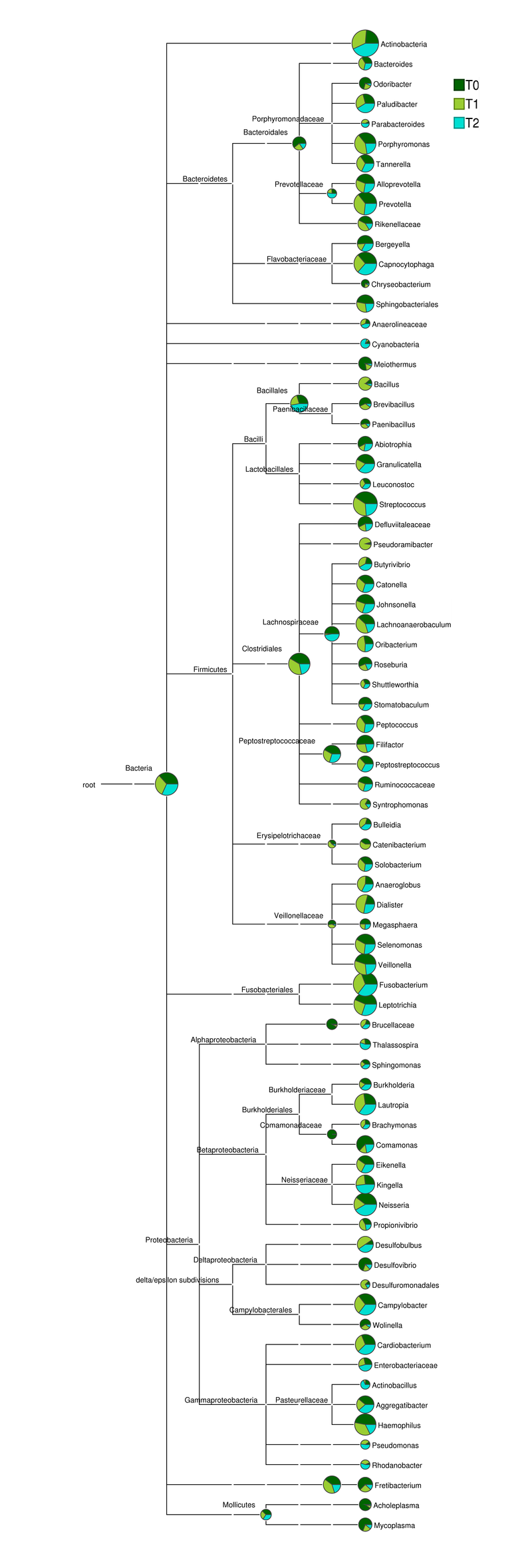

Supplement: Figure S1 — The larger the area of the pie chart at each level, the greater the bacterial abundance. Different colors represent different time points. The larger the colored sectorial area within the pie chart, the greater the bacterial abundance at the corresponding time point. [file peerj-06-4207-s001.png]
